# Participation of National Medical Associations in quality improvement activities - International comparison and the Israeli case

**DOI:** 10.1186/2045-4015-3-14

**Published:** 2014-04-25

**Authors:** Baruch Levi, Malke Borow, Michelle Glekin

**Affiliations:** 1 The Israeli Medical Association (IMA), Ramat Gan, Israel

## Abstract

**Background:**

Many countries have devoted considerable efforts in an attempt to improve the performance of their health care systems. National Medical Associations (NMAs), along with other stakeholders, play a part in the promotion of such activities. The purpose of this paper is to explore the nature and level of participation of NMAs in activities of quality improvement in medicine, with a specific emphasis on Israel.

**Methods:**

The authors conducted a survey among NMAs around the world inquiring as to their involvement in three central aspects of quality improvement: clinical guidelines, quality measurement and continuing medical education (CME). In addition, they conducted a review of the literature in order to gather more information and complete the data collected in the survey. The findings were processed and analyzed comparatively.

**Results:**

Most of the NMAs surveyed participate in quality improvement activities at least to some extent. NMAs' main involvement is in the regulation of CME and they are involved to a much lesser extent in the preparation of clinical guidelines and in quality measurement. In Israel, the Israeli Medical Association (IMA) has a dominant role in both the preparation of clinical guidelines and the regulation of CME credits.

**Discussion:**

It is possible that the expertise maintained by the profession, coupled with the organizational power of the NMA as a union, is viewed as beneficial for regulating educational activities in medicine such as CME. Conversely, the issuing of clinical guidelines is usually regarded as a typical scientific activity, and therefore often rests in the hands of professional medical societies. Quality measurement is regarded as a distinctive administrative tool and is usually found in the province of governments. Based on the typology that we introduced in our previous paper, we discovered that the extent of NMAs’ involvement in quality improvement coincides with the mode of governance of the health care system.

**Conclusions:**

The nature and level of participation of NMAs in activities of quality improvement varies widely. Collaboration of NMAs in this field with other stakeholders is not uncommon, and may contribute to the further development of quality improvement in medicine.

## Introduction

There is growing interest in the various forms of task sharing between governments and other stake holders in health care systems, among them national medical associations (NMAs) [1-6]. Although an NMA may traditionally serve as a trade union, it may also take upon itself additional roles and serve as a professional standards setter, policy maker, ethical arbiter, disciplinarian, or some combination of the above.

In a recent IJHPR article, we explored the role of the NMA as regulator of the medical profession, and the division of labor between the association and the government [1]. In this article, we seek to document its place in another key area - that of quality improvement. This realm, like that of public policy which we hope to explore in a third and final article, is a later addition to the ambit of professional activity.

In this article, we seek to determine in which activities of quality improvement different NMAs participate, and to what extent. We refer to the same framework of governmental structure that we explored in our previous paper - corporatist, market or hybrid systems. We posit that the structure in each country influences NMA involvement in quality improvement in the same manner, although not necessarily to the same extent, as it does more traditional regulatory activities such as licensing and specialty training.

### The promotion of quality improvement - background and literature review

Quality improvement in medicine can be described as a general effort to create a system of scientific, organizational and administrative activities designed to promote the improvement of healthcare services. In recent decades, many countries have devoted considerable efforts in an attempt to improve the performance of their health care systems. These efforts can be seen in a range of scientific, organizational, administrative and financial activities grouped under the heading of “quality assurance” or “quality improvement”. Policy makers, health authorities, health providers, insurers, associations of physicians and other health workers, as well as private and public bodies, are involved in continued and complex processes whose purpose is to ensure the quality of clinical treatment in particular, and the function of the health system as a whole [2].

Governments around the globe no longer confine themselves to the role of passive payers. Their gradual awareness of issues of efficiency and effectiveness has led them to search for ways to “ensure value for money”. Through quality improvement activities, governments seek to derive maximum medical benefit from the health care system under existing budgetary constraints. In this way, the ultimate goal of these activities – improving public health – is accompanied by an economic objective: to curb soaring health costs, while making the system more efficient.

Following the rapid spread of quality improvement activities in Israel and worldwide, and in light of their enormous importance to the government in recent years, the issue of allocating responsibility for their implementation has been placed on the agenda, and with it, the question of the medical association's role in promoting these activities. Several reports and surveys performed by research institutes and international organizations like the World Health Organization (WHO) and the Organization for Economic Cooperation and Development (OECD) have addressed the issue of medical regulation and the division of responsibilities between stakeholders. These works indicate the large number of bodies involved in regulatory tasks and the complexity of the regulation of health care systems [2-4]. However, although many parties might engage in quality improvement, not all are equipped to do so in an optimal fashion.

Legido-Quigley and colleagues discovered that clinical quality schemes that include, *inter alia*, clinical guidelines, quality measures, information systems and audit processes, often involve the development of new organizational structures, processes, measurement tools or methods [5]. De Vries and colleagues, who examined the regulation of ten health care systems around the world, state that the bodies responsible for regulation and quality assurance of medical education vary from country to country [4]. In particular, they examine different approaches to continuous professional development (CPD) and conclude that they vary widely. In Spain and Germany, for example, CPD is one of the main areas of activities engaged in by the provincial medical colleges and regional chambers of doctors, which often provide training courses and related services. In South Africa, this responsibility is entrusted in the hands of the Health Professionals Council of South Africa (HPCSA), which is the key medical regulation body in the country [4]. A similar picture of diversity and complexity regarding the regulation of continuing medical education is present in the WHO report on regulation and licensing of physicians from 2005 [6].

Or investigated initiatives to better measure and improve performance of health care systems in four OECD countries (France, the Netherlands, New Zealand and Sweden), describing in her work the role of institutional arrangements as well as policy and management “levers” used to bring about change in this field. The difference in the countries' approaches to the issue of quality improvement can be illustrated in the following example: In the Netherlands, health care providers have traditionally borne the primary responsibility for controlling and improving the quality of services provided. They are directly responsible for developing quality control systems, with explicit norms and procedures, and the government plays an active role in supporting these self-regulatory activities through a national policy for quality management. The government also holds a national conference every five years with healthcare providers, financers, and patient organizations in order to evaluate improvements and create consensus for new activities. The national health policy stresses that quality management is the joint responsibility of health care professionals and management [3].

On the other hand, in France, the State has much more responsibility for assuring the quality and efficiency of both primary and secondary care, employing a number of tools such as hospital accreditation, financial incentives and sanctions, and mandatory guidelines. France is one of the few countries which tried to impose mandatory clinical guidelines for medical practice. Although self-regulation in France continues to work in traditional ways, with private physicians being paid on a fee-for-service basis and patients having free access to any physician, the government does involve itself in the profession's medical autonomy with regard to activities of quality improvement, mainly the development of clinical guidelines [3].

Improving health care quality through medical education is also on the agenda in different countries. In the USA, a shared educational initiative of the American Medical Association (AMA) and 11 medical schools is currently being implemented. Its main target is to shift the focus of medical education toward performance improvement, concentrating on chronic care, teamwork, population health, and community [7]. In Israel, courses on patient safety and health policy issues have been introduced to the curriculum of undergraduate medical students at the Hebrew University of Jerusalem [8]. In addition, continuing medical education (CME) programs are evolving globally in order to enhance health professionals’ knowledge and skills [9].

The efforts described above to map and clarify the various approaches and models of medical regulation have contributed tremendously to the understanding of global developments taking place in the area of quality improvement. Nonetheless, although random and sporadic references to NMAs can be found in current literature, we feel that more information regarding the role of NMAs in this issue should be gathered and systematically analyzed.

As stated in our previous work, the role of the NMA is multi-faceted. With the development and expansion of the medical world, most NMAs no longer view themselves solely as trade unions, if at all. Therefore, it is valuable to examine the extent and nature of their intervention in the improvement of health care systems.

## Methods

While the nominal definition of “quality improvement” offered in the previous section helps in clarifying its nature and boundaries in general terms, in order to achieve a more operational definition of “quality improvement” one must identify its more dominant and measurable aspects.

The literature on quality improvement deals with a variety of different and important activities intended to advance the quality of health care. Although improving the professional capacity of physicians and other health professionals is a most desirable goal, quality improvement efforts are not limited to this purpose alone. Some quality improvement activities focus on the organizational level, such as the accreditation of hospital departments or the computerization of patient records [3]. Others concentrate on patient education and promote patients’ active involvement in shared decision making and access to information [5]. Financial incentives, regulation and policy strategies such as changes in reimbursement schemes or setting licensure requirements, can also be regarded as strategies of quality improvement on a more systematic level [5,6].

Many quality improvement strategies have been institutionalized in healthcare systems around the world. Three of them are mentioned repeatedly in white papers and international reviews issued by the OECD and WHO, as well as by national governments and various bodies of research: clinical guidelines, quality measurement and continuing medical education [2,5,6,9].

It is important to mention, in the Israeli context, that the Division of Medical Policy of the Israeli Medical Association considers these three activities to be at the core of clinical medical practice and engages in their promotion, while encouraging physicians to take part in their development [10].

These three core activities can be defined as follows:

•Clinical Guidelines – systematically developed statements to assist practitioner and patient decisions about appropriate treatment in specific clinical circumstances [11].

•Quality measurement - a mechanism that enables the quantification of the quality of a selected aspect of care, by comparing it to an evidence-based criterion that specifies what is better quality [12].

•Continuing Medical Education (CME) – a set of educational activities that serve to maintain, develop and increase the knowledge, skills and professional performance a physician uses when providing services for patients, the public, or the profession [13].

The involvement of NMAs can take many forms. It can be direct or indirect. It can be manifested in declarations and the publication of position papers, or in more active participation in the public and professional discourse through lobbying or other forms of pressure. It can be limited to formal representation in steering committees, or it may take a wider approach, such as the initiation and organization of conventions and academic courses. It is safe to say that almost all NMAs are involved at least to some extent in quality improvement. Nevertheless, a more rigorous approach is needed in order to be able to address the issue in a quantifiable and analytical fashion.

Similar to the method used in our previous work, we created a scale of four degrees between zero to three, with which to measure the scope of involvement of NMAs in quality improvement. The scale reflects the aggregate number of activities engaged in by NMAs as the prominent or leading body, in cooperation with another entity or exclusively (for instance, through delegation of responsibilities). Sometimes the administration and the medical profession share responsibility for regulating these activities, but often it is possible to point to one of them as the principal authority, or at least as the most prominent– usually the body that initiates the activity, executes it or supervises its execution by others.

The higher the number on the scale, the greater that NMA’s involvement in quality improvement. We also categorized NMAs according to their involvement in each of the three activities examined, in order to determine in which they are more likely to participate.

A questionnaire dealing with the involvement of medical associations in the improvement of quality was distributed by email to the 102 NMA members (at the time) of the World Medical Association (WMA), with the assistance of the WMA staff^a^.

The questionnaire asked the following questions:

a. *Who is responsible for issuing clinical guidelines? Is it a governmental or medical-professional body (medical association/society/chamber)? What is the role of the medical association (if any)?*

b. *What is the role of the profession in the regulation of performance measurement? Is this led by a governmental or medical-professional body? Is the medical association itself involved in this activity?*

c. *Is Continued Medical Education (CME/CPD) voluntary or mandatory for physicians in your country? (Mandatory = linked to re-licensure/re-validation/renewal of license etc.)*

d. *Who is responsible for administering CME credits in your country? Is this run by the profession?*

Where no reference is given in the text, the information stated was obtained from one of the surveys received.

In addition, we conducted a review of the literature, using search engines such as Google, Google-scholar and PubMed, in order to gather more information and complete the data collected in the survey. The findings were processed and analyzed comparatively.

22 countries responded to the questionnaire and are included in the comparative analysis given below: Albania, Australia, Belgium, Canada, China, Croatia, Denmark, Ethiopia, Germany, Hong Kong, Iceland, Israel, Kazakhstan, the Netherlands, New Zealand, Serbia, South Korea, Spain, Sweden, Taiwan, United Kingdom, United States.

The validity of study results may be affected by the low response rate (22%). Although not uncommon in WMA surveys of this sort, this low rate may reflect a possible bias in that NMAs that are more involved in quality improvement or work in systems that promote quality improvement may be more inclined to participate in the survey than other NMAs. A more effective way to encourage associations to respond to such questionnaires should be considered.

Without wishing to dismiss the contribution and importance of employers, insurers, pharmaceutical companies and other health organizations, we shall focus in this document on the role of medical associations vis-à-vis the government and scientific bodies that operate independently in the regulation of quality improvement activity. However, it should be noted that we did not examine the actual application of these activities in practice in every country, as this task is beyond the scope of this article. Theoretically, an NMA may be involved in three activities out of three, but in practice, none of these activities has any significance in that particular health system. On the other hand, an NMA may be in charge of one activity only (for example, quality measurement), but that particular activity may play a significant role in the relevant health care system. In that case, it is not clear at all which NMA has a more significant role, practically, in quality improvement. This may be considered a major constraint of our work for those who wish to learn more about the actual scope of quality improvement activity in different health systems. Nevertheless, the prime goal of our study is to describe the formal division of responsibilities among the government, the NMA and the various scientific bodies with respect to the three tools of quality improvement described above. We feel that a study of this kind could offer a good starting point for further studies focusing on the division of responsibilities in practice, and correlating the extent to which they align with the bureaucratic frameworks depicted in this paper.

## Results

The involvement of NMAs in quality improvement will be examined according to two aspects. First, as shown in the following figure, NMAs are categorized according to their total participation in the activities examined. Later, the NMAs will be categorized according to the their participation in each of the individual activities, in order to determine in which activities NMAs are more involved. The tables will be followed by examples from several countries that participated in the survey (Table 1).

**Table 1:** The extent of participation of NMAs in quality improvement activities

**Degree of participation**	**Countries**
3	Germany, China
2	Belgium, Israel, Spain, Taiwan
1	Albania, Croatia, Hong Kong, South Korea, The Netherlands, USA
0	Australia, Canada, Denmark, Ethiopia, Iceland, Kazakhstan, New Zealand, Serbia, Sweden, UK

**Figure 1:**
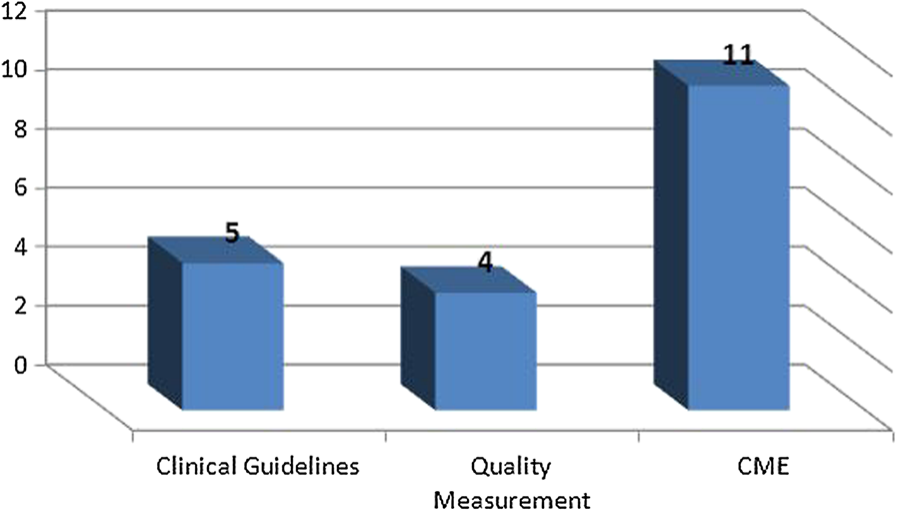
Categorization of NMA participation in quality improvement.

More than half of the NMAs surveyed have formal roles in quality improvement activities to varying degrees (12/22). Most notable are Germany and China, where the medical associations are involved in all three forms of quality improvement. Half of the 12 NMAs that participate in quality improvement, do so only to a minimal extent (degree of participation = 1), and are usually involved in CME^b^ (Figure 1).

The graph shows that half of the NMAs participating in the survey are involved in CME in their countries (11/22): Belgium, China, Croatia, Germany, Hong Kong, Israel, the Netherlands, South Korea, Spain, Taiwan, USA.

Only approximately one-fifth of the NMAs are engaged in the production of clinical guidelines (5/22) and quality measurement (4/22):

Clinical Guidelines: Belgium, China, Germany, Israel, Spain

Quality measurement: Albania, China, Germany, Taiwan

Responsibility for these two activities usually rests with bodies other than NMAs: scientific bodies (generally specialists’ associations) tend to focus on the development of clinical guidelines, while quality measurement is generally regulated by the government or a governmental agency.

As stated above we have separated quality improvement into three areas of work: clinical guidelines, quality measurement and continuing medical education. Each task will be reviewed independently, comparing the division of responsibilities between the profession and the government in a range of countries, based on the result of our survey and independent research.

### Clinical guidelines

The production of clinical guidelines has become an increasingly common part of clinical practice. There is great diversity among countries as to who is responsible for producing such guidelines and, even within certain countries, this practice is often conducted by several different bodies.

In Israel, as in many other countries, the responsibility for preparing clinical guidelines rests with professional scientific societies. These societies, also referred to as medical societies, are comprised of physicians in specific fields of specialization and focus on the scientific and clinical aspects of the profession. They may be, but are not always, part of the NMA. The Israeli Medical Association (IMA) acts as the umbrella association for its scientific societies and supports and encourages their activities. The IMA's Division of Medical Policy reviews the guidelines and assists with their preparation, publication and distribution to physicians in Israel. In addition, the Israeli Ministry of Health prepares clinical guidelines, in the form of circulars, through its advisory National Councils. The health funds also formulate their own internal guidelines.

Likewise, clinical guidelines in South Korea are mostly produced by the various scientific societies; however, the medical association noted that the government is increasing its input into the development of clinical guidelines.

The German medical community has a long tradition of self regulation, whereby the medical association and chambers perform an important regulatory role. The German Medical Association (GMA) is involved in the preparation of clinical guidelines through a number of its agencies, such as the Drug Commission, a scientific committee of experts in the field of pharmaceuticals, or the Scientific Advisory Board of the GMA, which consists of scientists representing all medical disciplines. The German Agency for Quality in Medicine (AQuMed), a non-profit organization established in 1995 by the GMA, and the National Association of Statutory Health Insurance Physicians, also participate in the preparation of clinical guidelines. These bodies prepare programs to promote quality in the health system, focusing on evidence based medicine (EBM), clinical guidelines, patient empowerment, treatment safety and quality management.

By way of contrast, in Iceland, the governmental Office of the Surgeon General is responsible for issuing clinical guidelines. Although the medical association was partially instrumental in establishing this service, it is not involved in any way in the day to day work.

In England, the National Institute for Health and Clinical Excellence (NICE) is responsible for developing clinical guidelines. They do so without the involvement of the British Medical Association (BMA). NICE was established in primary legislation as a Non Departmental Public Body in April 2013. The Department of Health instructs NICE to prepare guidelines in specific areas of health and assessments of medical technologies.

The Ministry of Health of the Republic of Kazakhstan is responsible for developing clinical guidelines in that country, in consultation with the National Medical Association. The governmental body develops draft guidelines and distributes these documents to the National Medical Association, which consults with its different branches before commenting.

It seems that NMAs are generally only marginally involved in the preparation of clinical guidelines, if at all. Examples like the IMA and the GMA are few, and in most cases the NMA is not considered a leading body in this aspect of quality performance activity.

### Quality measurement

There is an ever-growing demand from the public, healthcare providers, regulatory agencies and the government, for evidence based quality measurement. Quality improvement has become part of the everyday routine of many healthcare professionals [14].

In Israel, the Ministry of Health runs a national quality measurement program in the community with the cooperation of the four health funds [15]. In addition, the Ministry has begun a project to measure the outcomes of hospital care. This program is managed by a review committee appointed by the Director General of the Ministry of Health [16].

In the Netherlands, the Health Care Inspectorate (HCI), a government body, is responsible for developing measures of quality. The HCI works in conjunction with relevant professional bodies, including associations of specialists and general practitioners, as well as the Hospitals Association. Similarly, in New Zealand the government is primarily responsible for quality measurement through the Medical Council of New Zealand (MCNZ), a regulatory body. The MCNZ requires doctors to be fit to practice. District health boards measure and regulate performance through physician employment agreements or through funding requirements for general practice. The MCNZ Good Medical Practice publication provides guidance to doctors on the standards of practise which the MCNZ expects. The Health Practitioners Disciplinary Tribunal, the Council's Professional Conduct Committees and the Health and Disability Commissioner can (but are not required to) use this publication as a standard by which to measure professional conduct [17]. The medical profession also plays a role in the regulation of performance measurement. In particular, the Medical Colleges have various certification requirements for doctors, such as Cornerstone, an auditing tool for practices, established by the Royal NZ College of General Practice.

In Hong Kong, the performance of the medical profession is regulated and measured by the Medical Council of Hong Kong, a judicial body created under the Medical Registration Ordinance. The performance of the universities in Hong Kong is also under the scrutiny of the Education and Accrediting Committee of the Medical Council of Hong Kong.

While in the Netherlands, New Zealand and Hong Kong the medical profession (as opposed to the NMA itself) is in some way involved with their respective governments' regulation of quality measure, in Spain performance measurement is led by the government and the medical association is not involved at all.

In the United States there are several entities engaged in quality measurement – the government and its agencies, insurers, health service providers and scientific organizations. However, most activity takes place within the framework of joint initiatives by health service providers and government bodies [18]. Prominent among the government bodies is the Agency for Healthcare Research and Quality (AHRQ), which publishes an annual health index pursuant to the Healthcare Research and Quality Act 1999 [19]. The existence of a federal law indicates the importance that the United States places on health measurement, which includes measures of quality in medicine. The AHRQ works alongside the Center for Medicare and Medicaid Services (CMS), the National Quality Forum (NQF) and other bodies at the federal and state level responsible for promoting quality in the health system. Among their activities is the creation of financial mechanisms that link physicians’ pay to their performance [18]. The CMS is increasingly involved in quality measurement and has created several quality initiatives including: quality improvement, pay for reporting, and public reporting. CMS has also established a standardized system for developing and maintaining the measures used in its quality initiatives, known as the Measures Management System (MMS). This system is composed of a set of business processes and decision criteria that CMS funded measure developers follow in the development, implementation, and maintenance of quality measures.

The National Quality Forum (NQF) reviews, endorses, and recommends use of standardized healthcare performance measures and has endorsed the measures developed by the CMS Measures Management System [20]. The Physician’s Consortium for Performance Improvement (PCPI) is another entity, convened by the AMA, that works to develop evidence-based measures of clinical performance. It also provides resources for physicians to use as they become familiar with performance measurement. Members of PCPI include state medical societies, national medical specialty societies, AHRQ, CMS, the American Board of Medical Specialties and the Council of Medical Specialty Societies [18].

In Germany, the medical association oversees quality measurement. In September 2000, the German Medical Association (GMA), together with the Hospitals’ Federation and a number of insurers, set up the Institute for Quality and Patient Safety (BQS), which acts as an independent institute for the development of quality measures. In addition, the medical association and other bodies sponsor the “Forum Gesundheitsziele”, which works to develop national standards for health services. The GMA also works with the Agency of Health Technology Assessment (DAHTA) to publish reports on assessments of medical technologies. Another body that works in cooperation with the medical association on the subject of quality measurement is the Institute for Quality and Efficiency in Health Care, an independent body that studies the benefits and harm to patients following medical treatments.

In summary, the responsibility for quality measurement differs among the countries reviewed. In some cases non-governmental bodies, such as medical societies and NMAs, participate in the development of quality measures. In most cases, however, it is the government, sometimes via a designated entity, that is the most prominent stakeholder in this aspect of quality improvement.

### Continuing Medical Education (CME)

**Figure 2:**
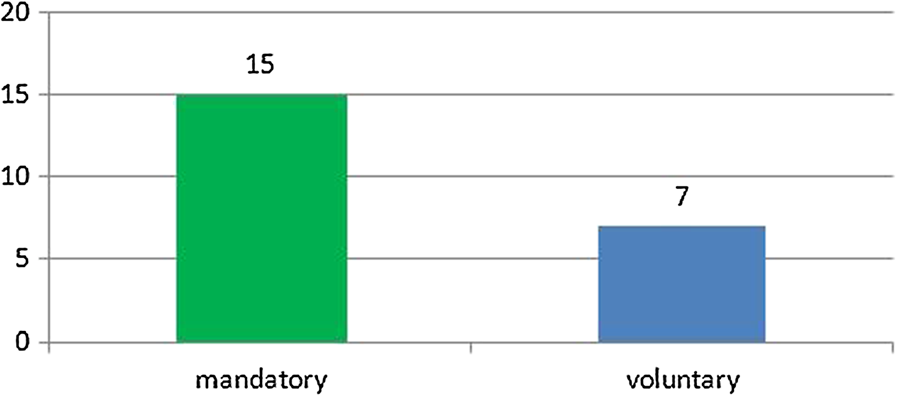
CME activity.

A great challenge for physicians today is staying abreast of the relevant material in their specific medical specialty. With new technology and scientific research, medicine is rapidly changing and medical education does not end with formal training [21]. The importance of continuing medical education is demonstrated by the many countries that have made it a mandatory requirement for their physicians (Figure 2).

As shown by the graph above, CME is mandatory in most of the surveyed countries, and is often linked to the renewal of physicians’ licenses^c^.

Mandatory CME: Albania, Australia, Canada, China, Croatia, Germany, Hong Kong, Kazakhstan, the Netherlands, New Zealand, Serbia, South Korea, Taiwan, UK, USA^d^.

Voluntary CME: Belgium, Denmark, Ethiopia, Iceland, Israel, Spain, Sweden

It is worth emphasizing that the role of NMAs in the area of CME is not limited to the provision of credits, but often includes the authority to decide which activities will be recognized for CME credits, their content and their contribution to the overall CME scheme. In other words, NMAs are not involved merely in the technical aspect of administering credits, but also help determine the quality standard of CME.

One question that needs to be addressed is the effectiveness of CME in improving the quality of care. Despite the generally low quality of evidence in existing studies, these studies suggest that CME is effective, at least to some degree, in improving the quality of care, especially when using interactive CME interventions such as conferences, courses, rounds, meetings, symposia and lectures [22-24].

In Israel, CME is undertaken on a voluntary basis. The health funds, hospitals, and scientific associations run professional refresher courses and workshops, and the IMA is responsible for granting credits for recognized activities. However, only a small minority of physicians routinely engage in formal CME activities.

The Royal Dutch Medical Association is responsible for CME in the Netherlands. Its subsidiary, the Central College of Specialists, manages continuing medical education and license renewal for specialists. The medical association controls and supervises these activities, and healthcare professionals earn credit for participation [25].

Since 1991, the Dutch Central College of Specialists has managed a system to renew the registration of specialist physicians, on behalf of 27 scientific associations. One requirement for renewal of registration is that every specialist in the country participates in 40 hours of recognized CME activity [2].

Similarly, in Germany, the German Medical Association's Regulation Framework for Continuing Medical Education serves as a model regulatory procedure for the State Medical Chambers. Continuing education activities are only recognized by the Chambers if they correspond to the requirements set out in the Medical Association's Regulation Framework [26].

In Taiwan, CME is mandatory and is run by the medical profession. The Taiwan Medical Association, Formosan Medical Association, Taiwan Association of Family Medicine, and the Taiwan Pediatric Association are responsible for administering CME credits in Taiwan. In the United Kingdom, CME is largely run by the scientific associations and institutions of the General Medical Council (GMC), a non-governmental regulatory body, with independent statutory status^e^. After lengthy policy consideration, continuing professional education is now a binding condition for renewal of licenses to practice medicine [27].

In Canada, "Maintenance of Competence" requirements, which are CME based, are administered through the educational bodies: the Royal College of Physicians and Surgeons of Canada and the College of Family Physicians of Canada. The Canadian Medical Association is not involved in these activities, although it communicates regularly with the organizations that are involved and makes the views of its members well known.

In Australia the practice is similar to that in Canada. CPD/CME credits are issued by the medical colleges, which have a framework for accrediting material eligible for CPD credits or “points”. These points may be used by the participating doctor towards his or her annual CPD requirements for ongoing College recognition and medical registration. The material is often delivered by College accredited education providers, rather than the College itself, and the content of the program must meet College accreditation requirements in order to qualify. The Australian Medical Association (State or Federal) can therefore deliver CME/CPD programs assuming they are College accredited providers.

With the recent introduction of the national medical registration, administered by the governmental Australian Health Professional Regulation Agency, physicians must report and record their participation in ongoing CPD as a requirement for medical registration. While the Australian Medical Association is not involved in performance measurement, it is considering providing a service to members by recording CPD.

In Serbia, CME is mandatory. The National Healthcare Council of the Republic of Serbia, which is elected by the National Parliament, is responsible for accreditation of CME programs, and members of Serbian Medical Chamber’s Assembly are involved in a special task group on CME. In contrast, in Sweden and Denmark, CME is not formalized at all and there is no requirement for medical professionals to partake in it.

In the United States, while CME is mandatory in the majority of states, Colorado, Indiana, Montana and South Dakota are the exception. The requirements of the different states also vary greatly, with the minimum hours of CME required ranging from 16 to over 100 hours [28]. The Accreditation Council for Continuing Medical Education (ACCME) is responsible for accrediting US institutions offering CME and their accreditation system is recognised as a national model by federal and state government agencies. The American Medical Association is one of the founding members of the ACCME, together with the American Board of Medical Specialties, the American Hospital Association, the Association of American Medical Colleges, the Association for Hospital Medical Education, the Council of Medical Specialty Societies, and the Federation of State Medical Boards of the United States. The seven founding members are also regularly involved with the regulation of the ACCME. Their duties include nominating individuals to the Board of Directors, providing input into ACCME’s strategic directions, and overseeing the ACCME actions and bylaws changes [29]. The AMA also provides some CME programs for physicians and offers a Physician's Recognition Award (PRA) which recognizes physicians who have earned an average of 50 credits per year from educational activities that meet AMA standards.

As seen from our survey findings, national medical associations are more likely to be involved in CME than in other areas of quality improvement. Nevertheless, in many cases they are not solely responsible for this practice. In many countries reviewed, such as Canada and Australia, CME is administered by educational bodies.

### Israel case study

As in other countries, health authorities in Israel invest considerable efforts in developing quality improvement activities. Their development has been accelerated during the last decade with the launching of quality measurement projects in primary care and the proliferation of clinical guidelines, both of which have seemingly gained the support of the medical community. On the other hand, mandatory CME has not been realized so far, despite the intention of the Ministry of Health. Although Israel is not considered unique in this regard, it has yet to join to the global trend of turning CME mandatory.

### Development of clinical guidelines

In Israel, the responsibility for preparing clinical guidelines rests with the scientific associations, which bear the responsibility for developing the theory of clinical work. The IMA supports this activity, and encourages its associations and physicians to write guidelines. The IMA's Medical Policy Division assists with the preparation, publication and distribution of these guidelines to doctors. The Division also reviews the standard of evidence on which guidelines are based, ensuring that they comply with the rules of Evidence Based Medicine (EBM) and cost effectiveness and that they do not conflict with other guidelines or instructions on the same subjects. The guidelines can be found both on the IMA website and that of the relevant scientific association, and bear the stamp of the IMA and of the association/s that participated in writing them. The health funds and hospitals that adopt the guidelines distribute them to their physicians.

It should be noted that the Ministry of Health also initiates the writing of guidelines through its circulars. Apart from the clinical guidelines of the IMA and its scientific associations, and the Ministry circulars, the health funds distribute their own internal guidelines.

Comprehensive research conducted in Israel in 2005 that dealt with the implementation of clinical guidelines among community physicians showed that the majority of physicians express a high to very high level of agreement with the assertion that clinical guidelines improve the quality of care (73%) and contribute to better clinical outcomes (62%). In addition, IMA’s position as a distributer of the guidelines among other stakeholders and its recommendations to physicians to make use of them, were speculated by the researchers as contributing to their acceptance by physicians. Nonetheless, it should be mentioned that Israeli physicians tend to adhere only partially to the guidelines, due, among other things, to structural barriers such as lack of time, workforce shortages and heavy workloads [30]. Nonetheless, given that this study was conducted almost ten years ago, it does not conclusively represent the attitude of today's physicians.

### Quality measurement

The main activity in the field of quality measurement in Israel has been carried out in community medicine for more than a decade. The Israeli program for quality measurement in community medicine began as a research initiative at Ben Gurion University in Be'er Sheba. The study was later expanded into a national program, in conjunction with the four health service providers, the support of the IMA, and under the auspices of the Israel National Institute for Health Policy Research. In 2004, the Ministry of Health declared the activity to be a national, permanent and established program, and in recent years it has taken over responsibility for its execution [15].

The program focuses on measurements in a number of important areas, such as preventive drug treatment for asthma, screening for breast and colon cancer, vaccinations against influenza and pneumococcus for adults, treatment of diabetes, and prevention and treatment of cardiovascular diseases. The aims of the national program are to improve the quality of community health services in Israel, and to provide the public and policy makers with information on this subject. At the same time, it has drawn criticism from doctors and associations, who argue that the program is managed by the health service providers in a way that does not always serve the patients’ interests. Many indicators in the program are performance measures that examine how physicians meet specific quantitative objectives (for example, the percentage of patients who are treated with medication for asthma or who have undergone mammography), without demonstrating the quality of the therapeutic process in terms of medical benefit (how effective is the medication in reducing asthma seizures? To what extent, if at all, has the increase in mammography contributed to breast cancer survival rates?). In addition, it is argued that health provider managements exert pressure on physicians to meet set targets, without considering the suitability of these targets to actual clinical reality [31].

Nevertheless, research from 2012 has shown that most primary care physicians (87%) in Israel felt that the monitoring of quality was important and two-thirds (66%) felt that the feedback and subsequent remedial interventions improved medical care to a great extent. The research indicates that only a minority of physicians oppose the monitoring program [32].

In addition to the community program, the Ministry of Health is currently planning a program to measure the outcomes of hospital care. This project began four years ago, and is managed by a review committee appointed by the Director General of the Ministry, and funded from the Ministry budget. The initial database contained surgical procedures from 2001 to 2008 in 26 general hospitals in Israel. As of 2010, it included repeat surveys of 24 departments of general surgery and 27 orthopedic wards. The activity involved a survey of outcome and process indicators used in quality measurement projects worldwide, such as infection of surgical wounds, rate of pneumonia among patients given artificial respiration, unplanned returns to the operating theater, complications during and after surgery, complications after release from the hospital, repeat hospitalization and others [16].

Discussion of the Israeli case would not be complete without reference to the IMA opposition to early efforts on the part of the Ministry of Health to impose quality measurement. In early 1994, the Ministry of Health established a Quality Assurance (QA) Division. The IMA requested to be included in the policy making and instructed its member physicians not to participate in surveys carried out by the Division. The Ministry's ability to carry out surveys and impose quality standards was thus severely limited. The IMA's concern was that without proper safeguards of confidentiality, the process would be used to harm the physicians, rather than improve quality. Indeed, in 1995 a scandal erupted when the results of quality measurement surveys were leaked to the press. The surveys indicated controversial results of excessive mortality rates in heart surgeries in certain hospitals. As a result of the publication, the Ministry of Health stopped the comparative measurements and reduced the activity of its QA division [33].

In 1997, a treaty was signed between the IMA and the Ministry that, among other provisions, stated that issues relating to quality surveys would be decided between both parties. Officials in the Israeli health care system claimed that this cooperation between the ministry and the IMA “buried” the quality surveys initiative [34]. The IMA later maintained that the treaty was not adhered to, and, in addition, other QA activities continued without the input of the IMA. Therefore the treaty was essentially impotent. Nonetheless, the IMA stands behind the importance of QA activities, provided they do not result in injury to the physicians.

### CME

Doctors in Israel are not currently required to renew their licenses, but there is a non-binding process of professional updating within the health funds and hospitals, and as an initiative of the scientific associations, which run professional refresher courses and workshops. The IMA grants credits for recognized activities, but they are not linked to renewal of licensing/ registration at this stage.

It should be noted that in June 2008 the Ministry of Health announced its intention of promoting re-registration for doctors, by requiring them to register with the Ministry every few years as a condition of practicing medicine. According to the media, the ministerial committee discussing this subject raised a number of options– from purely administrative registration to the possibility of pre-conditions for license renewal, including exams, quality control and reporting of complaints and negligence claims [35]. This announcement aroused the opposition of the IMA and as of now CME is still voluntary in Israel and only a small minority of physicians engages in this activity routinely, due to lack of positive or negative incentives to do so.

We see that the IMA is responsible for the administration of CME credits and for the preparation and proliferation of clinical guidelines, with the collaboration of the medical societies. On the other hand, it has no apparent formal role in the ongoing project of quality measurement in primary care, although its support was obtained in order to ignite the move at the beginning of the millennium. In addition, it seems there is extensive physician support for quality measurement and the issuing of clinical guidelines, as physicians acknowledge their importance to the improvement of clinical care. It can be assumed that this support has a positive effect on the feasibility of these activities and enables their continuation and further development.

## Discussion

One of the most noteworthy findings of the survey we conducted is that NMAs tend to focus on CME more than on other aspects of quality improvement.

Why are NMAs involved in CME predominantly more than they are involved in other areas of quality improvement? Should they be taking a more active role in other quality improvement activities?

One may speculate that the role of NMAs in CME activities is linked to their position in the previous stage of medical education – specialty training. In our previous work on regulatory tasks we examined health systems in five countries: Germany, Israel, the Netherlands, USA and the UK and compared the involvement of NMAs in specialty training, among other areas [1]. The research revealed that in three countries (Germany, Israel and the Netherlands) the NMA is the body responsible for specialty training. Thus it seems no coincidence that in these three countries the NMAs also play a central role in CME.

In the UK, on the other hand, CME credits are administered by the Medical Royal Colleges and not by the NMA, which similarly holds no responsibility for specialty training. The AMA is an exception on this matter, being the only NMA among the five examined that refrains from regulating specialty training on one hand, but fulfills a prominent role in CME on the other hand. In Israel and in the USA, the NMA administers the recognition and accreditation of educational activities necessary for the completion of learning cycles (for example, in Israel, the accumulation of at least 200 CME points in every 2 years). Despite the difference in the standing of the CME system in both countries (mandatory and linked to re-licensure in most states in the USA and completely voluntary in Israel), it seems that the position filled by the NMA in both countries with regards to CME is similar, and may reflect the dominance of NMAs in the regulation of educational processes around the world.

In light of these findings, it seems reasonable to assume that the same logic that drives governments to delegate the regulation of specialty training to the NMAs, leads them also to entrust NMAs with the regulation of CME; the expertise maintained by the profession, coupled with the organizational power of the NMA as a union, is apparently regarded as a useful tool in regulating educational activities in medicine, and, as stated in our previous work, “*setting the limits to governmental involvement in ’higher’ levels of medical professionalism”*[1].

Whereas the regulation of CME comprises both scientific and organizational aspects, the issuance of clinical guidelines is mainly centered on “pure” scientific effort with lesser organizational intervention. The gathering of medical data, the evaluation of evidence and the careful elaboration of clinical recommendations are considered sequential stages of a scientific process best left to experts. As opposed to CME programs, the use of clinical guidelines is not usually linked to a formal regulatory mechanism and is not routinely supervised or systematically organized as a mandatory activity. This may serve to explain why so few NMAs are involved in clinical guidelines, and why they are instead left largely to scientific societies and colleges, with or without the involvement of health ministries or agencies.

Israel is one of the few countries where the NMA is involved in the issuing of clinical guidelines. A possible explanation for this is the structural relationship between the IMA and the medical societies, wherein the societies are actually part of the IMA hierarchy, with no formal independent standing or legal status of their own. The symbiosis between the IMA and the medical societies in the realm of clinical guidelines can thus be perceived as an extension of the organizational structure of the medical community in Israel, where the IMA is comprised of the scientific bodies of physicians and plays a significant role in scientific activities. Conversely, quality measurement is an activity of a more administrative nature. Governments and employers accumulate information and process and compare clinical, statistical and economic data such as mortality and morbidity rates or volume of clinical procedures in the health institutions they operate. This systematic process may produce useful scientific findings that can be later translated into new clinical recommendations, but its main target is to improve the workings of the health care system, sometimes by providing an incentive or imposing a sanction on the healthcare provider.

Moreover, performance and health outcomes do not depend solely on the activities of physicians. They are first and foremost dependent on a set of factors that shape the workings and determine the capacity of the healthcare system as a whole, such as infrastructure, equipment, financial investments and organizational structure. The performance of the individual physician is derived, among other things, from these factors, and should be measured and referred to within this context. Therefore, quality measurement is usually dominated by the government or by agencies acting on its behalf, and not by the medical profession, be it scientific societies, colleges or NMAs (Table 2).

**Table 2:** The scope of compatibility between involvement in regulatory tasks and participation in quality improvement activities

**Country**	**Mode of Governance**	**Degree of involvement in regulatory tasks**	**Degree of participation in quality improvement activities**
Germany	Corporatist	3	3
Israel	Hybrid	1	2
Netherlands	Hybrid	1	1
US	Market	0	1
UK	Entrenched command and control	0	0

In the table above we tried to examine to what extent involvement in “traditional” regulatory tasks (licensing and registration, postgraduate training and physician disciplinary measures) coincides with participation in the more innovative activities of quality improvement.

It seems that the general pattern of involvement is preserved in the group of countries we previously studied. In the typical corporatist German health care system, the medical chambers are heavily involved in all 3 aspects of quality improvement, as they are in the case of the traditional regulatory tasks. Accordingly, in the hybrid systems of the Netherlands and Israel, there is a low to medium degree of involvement. A low to no involvement in quality improvement is demonstrated in the case of the USA and the UK, where local NMAs are only marginally, if at all, involved in traditional regulatory tasks.

Finally, we would like to briefly address the question of whether NMAs should take a more active part in additional quality improvement activities.

One of the main conclusions stemming from the international review performed by Or is that self-regulation of clinical care remains a vitally important institution in all four countries reviewed. By the same token, the experience of all four countries suggests that self-regulation can benefit from some external regulation and financial support from governments in advancing quality improvement activities. Active collaboration between medical professionals and policy makers/managers appears to be important for any policy. Clinical guidelines provide a clear example of this conclusion. The review indicates a somewhat disappointing experience that France has had with Regulatory Practice Guidelines (Références Médicales Opposables - RMOs). RMO is a unique initiative introduced by the French government in the 1990s, which tried to impose mandatory clinical guidelines on physicians in order to control medical practice. RMOs, prepared by a governmental agency, did not win the approval of the majority of French physicians, who viewed them as an instrument to reduce costs [3]. The physicians’ declining interest in RMOs led to a change in the regulator’s attitude towards the implementation of clinical guidelines. Control and sanctions gave way to collaborative efforts such as educational programs and campaigns, in order to disseminate clinical guidelines among physicians and enhance their acceptance by them. These efforts are usually well received by health professionals and there is evidence of their effectiveness in the treatment of type II diabetes and the use of antibiotics [36]. The French experience suggests that guidelines that do not have clinical ownership may ultimately fail, especially when imposed on physicians by the government without deliberating or collaborating with them.

We believe that the Israeli experience described above supports Or’s arguments, at least to a certain extent. Wider collaboration between the Ministry of Health, the employers and the IMA, as the representative body of the medical community, may enhance physicians’ confidence in the quality measurement project led by the Ministry and the HMOs, and would help in creating wider consensus regarding the method and indicators chosen. Contrary to France, clinical guidelines in Israel are prepared and distributed by the medical associations and the IMA, and as noted previously, they achieve relatively high credibility from Israeli primary care physicians.

Following Or’s work, we cautiously suggest that the fact that clinical guideline activity in Israel is almost solely in the hands of the profession, with the assistance and the organizational approval of the NMA, may have contributed to the acceptance of the guidelines and positive perceptions within the medical community. Therefore, it seems that further involvement of NMAs in quality improvement, through collaboration with other stakeholders, namely the government and the employers, can contribute to the development and implementation of this important task.

## Conclusion

In this article we set out to explore the role of NMAs in a key area of medical policy: quality improvement. We have found that the extent of their participation in quality improvement activities widely varies. Some NMAs are not involved in any of the three activities examined, but most of them participate in them at least to some extent. There seems to be compatibility between the scope of involvement of NMAs in regulatory tasks and their participation in quality improvement activities, whereby NMAs in corporatist health care systems tend to be more involved in the regulation of both more traditional tasks as well as in more innovative activities, namely quality improvement.

As to the nature of NMAs’ involvement in this area, it seems that NMAs are mainly in charge of the regulation of CME. We speculate that the expertise maintained by the profession, coupled with the organizational power of the NMA as a union, is regarded as a useful tool in regulating educational activities in medicine, in the same way NMAs in many countries are also responsible for postgraduate training. The issuing of clinical guidelines, on the other hand, is usually regarded as a typical scientific activity, less administrative in its nature, whereas quality measurement is regarded as a distinctive administrative tool. Therefore these two activities usually rest in the hands of medical societies and governments respectively.

As mentioned before, it should be noted that this work is limited to the examination of the formal roles that the NMAs perform, according to the structured division of responsibilities between them and other stakeholders. It does not deal with the scope of implementation of these activities in practice. Further efforts should be made in the future in order to examine this subject.

As discussed above, in Israel, the IMA is involved in the issuing of clinical guidelines in collaboration with its medical societies and it is also in charge of the regulation of CME credits. We suggest that the fact that activity regarding clinical guidelines in Israel is almost solely in the hands of the profession may have contributed to their acceptance and positive perceptions within the medical community, although no doubt further efforts should be made in order to strengthen the position of the guidelines in the clinical decision making process of medical practitioners. Therefore, it seems that further involvement of NMAs in quality improvement through collaboration with other stakeholders, namely the medical societies, the government and the employers, can contribute to the development and implementation of this important task.

## Endnotes

^a^The questionnaire also included questions regarding the regulation of the medical profession, which was the locus of our previous work, and questions regarding public policy which will be discussed separately in another work.

^b^There do not seem to be particular differences between “developed” and “developing” countries or between OECD and non-OECD countries regarding the degree of participation of NMAs in quality improvement or the kind of roles they tend to take upon themselves.

^c^The voluntary systems include those of Denmark and Sweden, although in both countries there is no official CME system. The medical associations and other bodies hold or approve courses for ongoing medical education, although there is no formal mechanism of credits.

^d^In the United States, while CME is mandatory in the majority of states, this is not the case throughout the country.

^e^In Britain, CME is sometimes called Continuing Professional Development (CPD). This term usually refers to an official study program and is more rigid than CME, which puts the emphasis on scientific content and is arranged by the scientific associations.

## Competing interests

The authors declare they have no competing interests.

## Authors’ contributions

The authors jointly conceived of the concept and design of the paper. MG focused primarily on data collection and drafting the empirical sections of the paper. BL focused primarily on the methodological and theoretical sections of the paper. MB was primarily responsible for data integration and editing. All authors read and approved the final manuscript.

## Authors’ information

Baruch Levi is a researcher of health policy at the Israeli Medical Association. He holds a BA in Economics and International Relations from the Hebrew University in Jerusalem, an MA in Political Science from Tel Aviv University and is currently working on his PhD about the effects of economic policies on public health.

Malke Borow is the director of the Division of Law and Policy at the Israeli Medical Association, which covers the fields of law, health policy and international relations. She holds a BA in psychology from Queens College/CUNY and a JD from Columbia University in New York.

Michelle Glekin is an International Relations Officer at the Israeli Medical Association. She holds an MA in psychology from the University of Edinburgh.
